# Brain-computer interface commercialization

**DOI:** 10.1186/s12984-025-01811-9

**Published:** 2026-01-29

**Authors:** Jackson Powell, Anson Zhou

**Affiliations:** 1 School of Medicine, Stanford University, Stanford, USA; 2 Medical Scientist Training Program, Stanford University, Stanford, USA; 3 Graduate School of Business, Stanford University, Stanford, USA

**Keywords:** Brain-computer interface, Commercialization, Rehabilitation technology, Spinal cord injury, Retinal prosthesis, Medical device market, Regulatory strategy, Physician adoption

## Abstract

**Background:**

Brain-computer interfaces (BCIs) have emerged as powerful tools to restore neurological function lost due to injury or degeneration. Despite scientific advancements, successful commercialization remains challenging. This article analyzes two rehabilitative BCIs—the Argus II retinal prosthesis by Second Sight and the ARC-IM spinal cord stimulation system by Onward Medical—as case studies to highlight critical factors influencing their commercialization outcomes.

**Main text:**

The Argus II, initially heralded as groundbreaking, restored partial vision to patients blinded by retinitis pigmentosa through direct retinal stimulation. Despite clinical success and initial regulatory approvals leveraging reduced barriers via humanitarian device exemptions, it struggled commercially due to an insufficient patient population, prohibitive manufacturing costs, and high procedural and postoperative rehabilitation costs. Physician adoption barriers, driven by the unfamiliar surgical procedure and reimbursement challenges, further limited its scalability, ultimately leading to market withdrawal. Conversely, Onward Medical adopted a phased commercialization approach with its ARC systems targeting spinal cord injuries. Initially releasing the non-invasive ARC-EX device, Onward built clinical traction and provider relationships, strategically fostering familiarity to minimize early risk and regulatory burden. Leveraging established spinal cord stimulation technology widely familiar to physicians, Onward positioned its invasive ARC-IM system more favorably for broader adoption. Despite remaining challenges, such as surgical complexity, postoperative rehabilitation infrastructure, and cost, this methodical strategy may significantly mitigate commercial and clinical barriers.

**Conclusion:**

Comparative analysis of Argus II and ARC-IM underscores that thoughtful commercialization strategies emphasizing phased product introduction, leveraging predicate technologies, and aligning closely with established clinical practices are essential to translating BCI technologies from scientific milestones into sustainable clinical solutions.

## Background

As more companies are on the verge of commercializing, the brain-computer interface (BCI) market is ever growing, with estimates putting the size at greater than $6 billion by 2030 [1]. The vast majority is accounted for by BCIs designed to regain function lost after nervous system injury or degeneration. Such BCIs can be broadly classified as *rehabilitative* (restoration of function, such as stimulation inducing neurons to regrow) or *assistive* (replacement of function, such as controlling a bionic limb), and can be non-invasive or invasive. Here we discuss two scientifically successful rehabilitative BCIs–both of which require implantation and electrically stimulate neurons to restore the function of broken circuits. The first, the Argus II by Second Sight, restores partial vision to blind patients. The second, the ARC-IM by Onward Medical, improves limb function in spinal cord injury patients. While both devices present advances that seem like science fiction brought to reality, the Argus II eventually failed in the market. Through thoughtful commercialization strategy, Onward Medical may avoid the same pitfalls that Second Sight fell into, laying a roadmap for successful BCI commercialization.

BCI commercialization follows many of the same principles as broader medical device commercialization. However, when they fail, the outcomes are particularly devastating—patients lose their ability to interact with the world around them. This risk should not dissuade companies from attempting to help these in-need patients, but rather should prompt finer discussion around strategies for effective and successful commercialization. Here we explore such strategies for BCI commercialization.

## Main

### Vision restored and lost again

Second Sight Medical Products illuminated possibilities in 2011 with the approval of Argus II Retinal Prosthesis in the European Union, followed shortly by the FDA’s approval under the Humanitarian Device Exemption (HDE) in 2013. The system could treat patients with blindness due to retinitis pigmentosa—a genetic disease where photoreceptors become nonfunctional [2]. The technology itself was a remarkable feat of engineering and neuroscience. By sitting atop the retina and directly stimulating the optic nerve from its 60-electrode array, patients regained the ability to see simple structures. Though, stimulation of the optic nerve and not bipolar cells meant that the device lost some stimulation precision, and patients only regained the ability to see in black and white. While far from complete restoration, it was enough to have a noticeable impact on patients’ lives. For example, a patient described the ability to visualize the form of a singer on stage at a concert and to see the stripes of a crosswalk again [3]. Further, some patients reported that their vision continued to improve after implantation, demonstrating that the technology itself was certainly a scientific success. Yet, driven by years of financial struggles, Second Sight began phasing out the Argus II in 2019 and paused all technical support for existing users in 2020, unfortunately leaving some blind once again [4].

### Balancing level of regulatory burden and a commercially sustainable patient population

The selection of retinitis pigmentosa as Argus II’s first indication was certainly driven in large part by the reduced regulatory barriers for rare diseases without a cure. With a US incidence of 82,500 to 110,000, Argus II qualified for the FDA’s HDE pathway by focusing on a subset of the worst affected patients [5], avoiding millions in costs by sidestepping otherwise necessary large-scale clinical trials and premarket approval requirements [6]. The typical clinical efficacy burden of proof was reduced to only proof of safety and a *probable* benefit. For a relatively small medical device startup looking to bring its first successful product to market, this was a smart strategy to maximize the speed and probability of commercialization. But despite a goal of reaching 2,000 patients within the first 5 years after approval [7], nearly a decade passed before only 350 patients had received the implants globally, generating ~$32 M in revenue [8]. After nearly $300 M in investments and grants over two decades, and no sign of revenue scaling, the selection of retinitis pigmentosa became more a barrier than an opportunity. The small patient population coupled with the threshold required to qualify—physically, socially, and psychologically healthy for surgery and rehabilitation—proved difficult to surmount [9]. Second Sight eventually attempted a classic strategy of indication expansion and launched a trial for age-related macular degeneration (AMD) in 2015, increasing its potential market multi-fold [10]. However, this came too little too late, and the product was discontinued prior to ever receiving approval for AMD in the US. In Second Sight’s 2020 financial report, following the rollback of the Argus II, they wrote, “We have not been profitable to date and expect our operating losses to continue for the foreseeable future; we may never be profitable” [11].

### New technologies increase costs and complicate the value chain even after approval

Second Sight, after FDA approval, achieved a significant milestone by successfully negotiating reimbursement coverage of $150,000 with the Centers for Medicare and Medicaid Services (CMS)—substantially higher than that of existing neuromodulation technologies [12]. Similarly, following Conformité Européenne (CE) mark approval in the European Union, economists argued that in Europe, the technology met acceptable cost-effectiveness levels and remained below the typical willingness-to-pay threshold [13]. These findings subsequently resulted in reimbursement program approvals in Germany (NUB), France (Forfait Innovation) and local/regional funding in the Tuscany and Veneto regions of Italy [14]. In 2018, the year before the discontinuation of the Argus II, Second Sight reported a gross margin of 29%. While the figure aligns with medical device industry norms, in the context of its excessive price point, it is indicative of a high cost of goods sold. Declining demand drove down sales volume, leading to potential margin compression [11].

As a first-of-its-kind product, the Argus II sought to establish a technical moat against others in the rehabilitative BCI space. However, its technical sophistication also presented a significant challenge in production. The device’s constituent components were designed by Second Sight, who acted as the original equipment manufacturer (OEM), including the electrode array and integrated circuit. The specialized nature of the components meant that high-cost contract manufacturing was required as opposed to more off-the-shelf elements [15]. This, combined with the low purchase volumes, further exacerbated margin compression. While this risk is difficult to mitigate with a device of this novelty, finding a balance between performance, convenience, and ease of manufacturing is critical to long-term commercialization.

### Convincing physicians to learn completely new procedures is challenging

The novelty of the technology created barriers to post-production scaling and adoption. Establishing relationships with eye centers and equipping them for implantation proved to be a challenging process. By 2015, nearly half of the eligible centers were still in Europe [16]. This was driven at least in part by the high costs beyond the device itself, coupled with slow reimbursement. The implantation procedure and subsequent rehabilitation costs exceeded the device’s price, adding another $350,000 to the total expense. Efforts to onboard more procedural partners were hampered as implanting surgeons often delayed additional procedures until reimbursement was secured [16]. The lack of Medicare Administrative Contractor (MAC) reimbursement approval meant that each use had to be evaluated on a case-by-case basis by Medicare Advantage providers [14].

Attracting provider buy-in also became a major barrier as Second Sight attempted to scale, driven by the unfamiliarity with the procedure and the lack of conviction surrounding substantive quality of life improvements. In a committee of special adviser ophthalmologists from the Royal College, the consensus was that fewer than 10% of specialists engaged in this area of work, and some advisors considered it the “first in a new class of procedure[s]” [17]. It is classically difficult to convince physicians to adopt new, complex procedures, and even more so in instances such as with the Argus II where there was little to no precedent [18]. In these instances, startups may benefit from partnering with established players in adjacent domains, in order to leverage capital, sales channels, and credibility for better chances of successful clinical adoption.

### Bridging the spine, grasping the market

Stimulating the spine to improve recovery after injury is novel in practice, but not in principle. Using electronics to stimulate the spinal cord is done tens of thousands of times a year for neuropathic pain and the basic design and hardware of spinal cord stimulators have existed for decades [19, 20]. Yet, Onward Medical adapted this technology to treat the untreatable: spinal cord injury, an ailment that affects 15 million people globally [21]. As it stands, Onward Medical’s flagship device, the ARC-IM, is undergoing a pivotal clinical trial [22]. The implanted device features brain wave recording, decoding and transmission to the spine, and stimulation of hotspots relevant for ambulation.

### Phased product commercialization for scalable impact

Unlike Second Sight, which targeted a specific patient population with a surgically implanted system from the outset, Onward Medical has taken a more gradual approach. Rather than launching the flagship ARC-IM—an implantable spinal cord stimulation device—as their first product, Onward first commercialized the ARC-EX, a non-invasive system. The ARC-EX trial, with 60 patients focused on upper limb function through the combination of transcutaneous stimulation and rehabilitation, provided a lower-risk entry point with encouraging clinical outcomes [23]. Granted marketing access by the FDA via the *de novo* pathway at the end of 2024, this allowed the company to gather real-world data, build relationships with rehabilitation centers, and establish a foothold in the market without the high regulatory and adoption hurdles associated with novel implanted devices.

ARC-EX’s target condition—spinal cord injury—affects a greater US population of 253,000 to 378,000. While severe spinal cord injuries still limit patient eligibility, the potential market is larger and the range of clinical symptoms addressed is wider. Spinal cord injury can cause incontinence, limit sexual function, and induce autonomic nervous system dysregulation [24, 25]. While both Second Sight and Onward Medical target patient populations in desperate need, this range of symptoms affords Onward Medical the chance to design a more versatile device. As new patents come from the research group, the potential upside for patients continues to improve [26–29]. By exploring adjacent applications such as Parkinson’s disease, Onward is positioning itself for future market expansion rather than locking into a single, restrictive indication [30].

### Leveraging predicate technology and existing frameworks for risk mitigation

Second Sight struggled with the Argus II’s technical novelty and associated high costs. Specialized components, costly manufacturing, and limited production kept margins tight and low demand prevented economies of scale. Onward Medical’s approach to commercialization is more sustainable. By first launching the lower barrier ARC-EX, the company can generate revenue while refining their higher-risk technology and scaling operational infrastructure. The ARC-EX acts as an intermediate device that can be used in rehabilitative settings with less severe injuries. Following up by commercializing the ARC-IM allows Onward Medical to capture a different, but overlapping patient population. Depending on the mode, location, and severity of injury, they can offer a different device to meet that patient’s needs. Additionally, this early commercialization helps to establish reimbursement pathways, de-risking the transition to the implanted ARC-IM system.

### Alignment with established procedures for easier clinical adoption

Physician adoption was a major hurdle for Second Sight. Onward Medical has structured its approach to avoid this pitfall. The ARC-EX requires essentially no procedure, allowing rehabilitation specialists to integrate the technology into existing care protocols. When moving to the ARC-IM, Onward benefits from the fact that spinal cord stimulator implantation for neuropathic pain is a familiar procedure for many neurosurgeons and trained pain specialists [31]. Unlike the Argus II, which had no real precedent in ophthalmology, the ARC-IM is an evolution of existing technology, making physician buy-in easier.

However, it is key to note that the ARC-IM still has key barriers to overcome. For example, the surgery, requiring both a laminectomy and a durotomy, may be too risky for many patients. It is unclear what proportion of paralyzed patients will be able to endure such a surgery, and how long the recovery periods will last. Furthermore, as the ARC-IM still relies heavily on rehabilitation, the post operative cost burden is likely to be high. Indeed, specialized centers and many physical-therapist hours will be required for each patient implanted. Therefore, there still may be difficulties in scaling adoption on the post operative side, as the infrastructure needed to support patients after implantation may not be present at scale [32].

## Conclusion

When neurotechnology companies go out of business, nonfunctional devices are left inside patients [30]. Thus, with each new company, ask: Who will be implanted? By whom? Is it worth it? The Argus II faced many challenges with insufficient upside, but lessons from it should not discourage attempts to commercialize academic feats, rather it should encourage more pragmatic approaches to commercialization.

Indeed, Second Sight is taking that second chance. After ceasing nearly all operations, in 2023 it merged with Nano Precision Medical and was re-spun out as Cortigent, focusing on the ORION visual cortical prosthesis to address blindness and marketing as the “next generation” following the Argus retinal prosthesis modality [33]. Having learned the scrupulousness of BCI commercialization, ORION has cast a wide net in its ongoing clinical trial, recruiting patients across a range of blind pathologies [34]. While the Argus II’s initial commercialization was not successful, perhaps the ORION, learning from past mistakes, will be.

These innovations emphasize a vital lesson for neurotechnology commercialization: scientific innovation must be paired with sustainable market strategies and clear patient-focused value propositions. By integrating these insights, future companies can better translate academic achievements into lasting clinical impact, safeguarding both patient welfare and technological advancement in the field.

## Data Availability

No datasets were generated or analysed during the current study.

## Abbreviations

AMD: Age-related macular degeneration
BCI: Brain-computer interface
CMS: Centers for Medicare and Medicaid Services
FDA: Food and Drug Administration
HDE: Humanitarian Device Exemption
OEM: Original equipment manufacturer
